# An Iatrogenic Model of Brain Small-Vessel Disease: Post-Radiation Encephalopathy

**DOI:** 10.3390/ijms21186506

**Published:** 2020-09-05

**Authors:** Rita Moretti, Paola Caruso

**Affiliations:** 1 Department Medical, Surgical, Health Sciences, University of Trieste, Cattinara Hospital, Strada di Fiume, 447, 34149 Trieste, Italy; moretti@units.it; 2 Neurological Clinic, Department of Internal Medicine and Neurology, University of Trieste, Cattinara Hospital, Strada di Fiume, 447, 34149 Trieste, Italy

**Keywords:** radiation-induced cognitive impairment, small vessel disease, oxidative stress and neuro-inflammation

## Abstract

We studied 114 primitive cerebral neoplasia, that were surgically treated, and underwent radiotherapy (RT), and compared their results to those obtained by 190 patients diagnosed with subcortical vascular dementia (sVAD). Patients with any form of primitive cerebral neoplasia underwent whole-brain radiotherapy. All the tumor patients had regional field partial brain RT, which encompassed each tumor, with an average margin of 2.6 cm from the initial target tumor volume. We observed in our patients who have been exposed to a higher dose of RT (30–65 Gy) a cognitive and behavior decline similar to that observed in sVAD, with the frontal dysexecutive syndrome, apathy, and gait alterations, but with a more rapid onset and with an overwhelming effect. Multiple mechanisms are likely to be involved in radiation-induced cognitive impairment. The active site of RT brain damage is the white matter areas, particularly the internal capsule, basal ganglia, caudate, hippocampus, and subventricular zone. In all cases, radiation damage inside the brain mainly focuses on the cortical–subcortical frontal loops, which integrate and process the flow of information from the cortical areas, where executive functions are “elaborated” and prepared, towards the thalamus, subthalamus, and cerebellum, where they are continuously refined and executed. The active mechanisms that RT drives are similar to those observed in cerebral small vessel disease (SVD), leading to sVAD. The RT’s primary targets, outside the tumor mass, are the blood–brain barrier (BBB), the small vessels, and putative mechanisms that can be taken into account are oxidative stress and neuro-inflammation, strongly associated with the alteration of NMDA receptor subunit composition.

## 1. Introduction

Ions derived from electrons’ ejection from either atoms or molecules, secondary to different stresses such as high temperature, electrical discharges, or electromagnetic and nuclear radiation [1,2], are the basis for radiation.

Radiation-induced brain injury is classified as acute, early delayed (subacute), and late-delayed reactions based on the timing of the onset of symptoms [3,4,5]. Acute injury, occurring 48 h to several weeks after the end of the radiotherapy cure, is characterized by general symptoms such as fatigue and sleepiness, drowsiness, and central symptoms such as memory loss and local, and not irreparable, demyelination. On the other hand, the definite injuries occur from six months up to several years after radiotherapy; the demyelination is overwhelming and irreversible and ends in white matter necrosis [6]. The late-onset radiotherapy (RT) damages are irreversible and often fatal, especially when patients undergo chemotherapy together with radiation, (whole-brain RT (usually 4000–4500 cGy) plus a focal tumor boost [7,8]; the potentiating effect of nitrosoureas, chemotherapy on the neurotoxic effect of RT [9,10]; with methotrexate [11], temozolomide [12], or rituximab [13]). Although there have been significant developments in understanding the pathophysiological mechanisms of radiotherapy-induced brain damage, limited information on the etiology of radiation-induced damage to healthy brain tissue is currently disposable.

Radiation causes damages directly on DNA strands and on RNA and destroying all the cellular bilayers, by inducing calcium inflow, stimulating apoptosis by mechanisms not completely understood [14,15]. Preclinical molecular studies have clarified that radio-induced brain damage is correlated with radiation exposure, the total administered dose, dose per fraction, and the duration of irradiation [16].

Many studies have failed to acquire a safety dose/brain–volume–target ratio, and the general acquired accepted data conclude that the maximally tolerated dose for boards of maximum diameter comprised in between 31 and 40 mm should be 15 Gy, whereas it could be of 18 Gy for boards of diameter in between 21 and 30 mm. The main results are, in clinical practice, wholly discordant, and if anything definite has been written, due to the disproportionate differences in the definitions of the target volume, the determination of the toxicity, it is necessary to protect critical brain structures from the RT beams, depending on the type and length of clinical follow-up [17]. The European Platform of Cancer Research (EORTC) Radiotherapy Cooperative Group, the EORTC Brain Tumor Group, and the Brain Tumor Working Party of the MRC (UK) participated in this long determining study [17]. The results were discordant, since the publication of the interim results, in which two factions were outlined: one which supported and the one which condemned RT. The non-believers in RT supported RT’s side-effects and did not justify a precocious RT, suggesting that delayed RT might be equally effective [12,17].

This is why new techniques have been implemented to ameliorate RT toxicity risks, usually given by external beam RT. Many centers now possess many different other potential systems, such as intensity-modulated radiation therapy (IMRT). This technique permits to shape and adjust beams’ intensity and direction. The potential benefit is obvious, i.e., the total or the partial spearing of the optic nerve, the brain stem, and the pituitary gland. On the other hand, this procedure permits to implement radiation intensity toward the tumor target. Stereotactic radiation therapy is external beam radiation directed through the tumor, also called the treatment field, via a 3D model, previously created by CT or more often via an MRI picture. Finally, the more non-conventional and modern technique is conformal proton beam radiation. Protons are charged particles and have a very rapid energy loss in the last few millimeters of penetration. Therefore, it is possible that a sharply localized peak of dose, known as the Bragg peak, is directly related to the initial energy of protons. Therefore, the desired dose can thus be precisely placed, with limited radiation dispersion. Unfortunately, proton beams are not easily obtained and are quite expensive.

Nevertheless, one of the most recent works on the topic by EORTC leaves us in a still open debate [18], and two studies showed that the quality of life after brain RT decreases and suggests tumor consequences as for the RT side-effects [19,20]. Irreversible cognitive impairment as an RT consequence is, unfortunately, well demonstrated by many different studies [11,12,21,22,23].

## 2. Our Recent Data

### 2.1. Subjects

We expanded a recently published study [12]; we enclosed the results of 114 cases (45.2 ± 3.7 years old) of primitive cerebral neoplasia, surgically treated; all of them underwent radiotherapy (Group A). They were enrolled by the Complex Neurological Case Section, to be studied and then prepared for neurosurgery. Patients with focal Broca or Wernicke’s area brain tumors were excluded due to major, confounding linguistic impairment.

Control subjects were 190 men and women, (72.3 ± 2.3 years old) (group B), diagnosed with subcortical vascular dementia (sVAD) entering the Neurological Unit of the University of Trieste. Their Mini-Mental State Examination (MMSE) scores were of at least 16, and they satisfied the fifth edition of the Diagnostic and Statistical Manual of Mental Disorders (DSM-V) for dementia, recruited from 1 June 2010 to 1 June 2016. They satisfied the criteria for probable VaD following the NINDS-AIREN criteria [24]. A patient was diagnosed with subcortical VaD (sVaD) when the CT/MRI scan showed moderate to severe ischemic white matter changes [25,26,27,28,29,30,31,32] and at least one lacunar infarct. All patients underwent a standardized baseline assessment that included a detailed history, a physical examination, laboratory tests, and neuropsychological assessment. Visits were scheduled to occur every three months after the beginning of the RT treatment and were equivalent for both groups.

This study was conducted following the Declaration of Helsinki and the Ethics Guidelines of the Comitato Etico Ospedaliero Univeristario Regionale (CEUR), point 4. Written informed consent was obtained from all participants or their responsible caregivers before the study.

### 2.2. Methods

We followed our previously published criteria of testing selection [11,12], even though many different schemata have been proposed for a neuropsychological evaluation of RT encephalopathy [33,34]. This study’s primary purpose remained to compare the executive behavior and motor scores in the two studied groups, one of de novo RT-treated patients and one of sVAD patients. To implement the study of executive functions we employed MMSE [35], Digit Span Forward and Backward [36], a three-minute phonological and semantic fluency test [37], as well as mental and written calculation test [38]; behavioral scales were chosen for the Apathy Score (AES-S and C) [39] and the Behavioral Pathology in AD Rating Scale, BEHAVE-AD [40]. We studied the gait and equilibrium with the Tinetti scale [41]. Handedness was determined by Briggs and Nebes test [42].

### 2.3. Statistical Analysis

Statistical analyses were performed using SAS^®^ software (version 16.2 SAS^®^ Software Inc, Cary, NC, USA). The difference in baseline characteristics between the RT and sVAD patients was assessed by the ANOVA test for the categorical variables; in case the ANOVA results were found to be significant, the multiple comparison analysis was also done by the Tukey test, to examine those groups which were significantly different for each other. Between group comparisons of changes from baseline were tested using the Marginal Homogeneity Test. Results are presented with standard deviations, and *p*-values are provided where appropriate.

### 2.4. Results

We followed 114 cases of primitive cerebral neoplasia (Group A) (Table 1). They underwent neurosurgery; the surgical operations were radical in 27% of cases; 32% of cases could be considered partially efficacious; and in 40% of cases surgery could be considered a stereotaxic biopsy. The final histological examination concluded 44 cases of glioblastoma multiforme; 26 cases of cerebral lymphomas (mild- or low-grade differentiation); 19 cases of grade-2 gliomas; 8 cases of craniopharyngiomas; 12 cases of II-III grade-astrocytomas; and 5 cases described as anaplastic patterns.

All of Group A was submitted to radiotherapy: the patients underwent fractionated external beam radiation therapy (from 20 to 65 cGy). Patients with any form of primitive cerebral neoplasia underwent WBRT: radiotherapists used the regional fields partial brain, encompassing each tumor, with an average margin of 2.6 + 0.7 cm from the initial target tumor volume. Therefore, in this study, we did not consider the size of the tumor/radiation field ratio.

We chose 190 sVAD patients which were Group B. All the patients could be thoroughly studied (mean age 72.3 ± 2.3 years). The diagnosis was based on historical information and neuropsychological assessment and was supported by structural (CT or MRI) imaging findings. The subsequent follow-up of subjects reinforced the clinical diagnoses in all cases. A neurologist (RM) revised all the imaging, employing the CT scans [29] or the MRI imaging scales [27,28,31,32] in sVAD patients.

All the surgery patients underwent two preliminary neuropsychological tests to identify an eventual decay of cognitive performances due to the tumor mass localization or the consequence of neurosurgery.

Then, both groups were examined at recruitment after radiotherapy, 3, 6, and 12 months after. At the end of the follow-up process (12.2 + 4.6 months), Group A characteristics were reported in Table 2. Twenty-six patients underwent a total RT dose <30 Gy; their histological profile could be summarized as eight craniopharyngiomas, ten grade-2 gliomas, and eight grade-2 astrocytomas. All of them underwent CT. Forty-four patients underwent a total RT dose of 30–45 Gy; their histological profile resulted as eighteen glioblastoma multiforme, ten cerebral lymphomas, nine grade-2-gliomas, four grade-3-astrocytomas, and three anaplastic patterns. Six patients underwent methotrexate (MTX) simultaneously, six patients underwent Rituximab and six underwent Temozolomide. Finally, forty-four patients underwent a total RT dose comprised between 45 and 65 Gy. Their histology was composed of twenty-six glioblastoma multiforme, sixteen cerebral lymphomas, and two anaplastic patterns. Ten patients underwent MTX treatment at the same time, 16 underwent Rituximab, and 26 with Temozolomide.

Twelve patients (45–65 Gy RT) died during the follow-up, and they were not taken into account for the final statistical analysis. A total of 102 patients were thoroughly studied. All the oncological patients underwent MRI scans after RT. The increase in white matter hyper-intensities on the T2-weighted images, markedly on the periventricular regions, was consistently evident from 30–45 to 45–65 Gy and were localized mainly in the internal capsule, the paraventricular frontal and basal ganglia areas.

At baseline, sVAD patients did worse in digit span, analogies, and semantic fluency (see Table 3).

Twenty-six patients who received a cumulative dose of radiation <30 cGy were compared to sVAD patients. Results demonstrated that sVAD patients after one year from the diagnosis were worse in phonological and semantic fluency, analogies, digit span backward, apathy (Table 4).

Forty patients who received a total dose comprised between 30 and 45 cGy manifested a global slowness of the cognitive process. They did worse than the sVAD Group after 12 months in phonological fluency tasks, analogies, and digit span scores. Their behavior deteriorated more rapidly than the sVAD group, in terms of apathy scores (Table 5).

Thirty-six patients underwent high doses of radiation therapy (45–65 Gy) and presented at 12 months a significant deterioration of all the tested performances (Table 6).

A marginal homogeneity test revealed a significant decrease in the performances in all the scores when compared to the higher to the lower RT doses (Table 7).

All the patients who received oral chemotherapy plus RT, 18 patients in the 30–45 Gy subgroup, and 44 in the 45–65 Gy subgroup more rapidly manifested gait disorders and more severe and rapid cognitive deterioration, associated with a loss of awareness, and were bed-ridden at the end of the follow-up.

### 2.5. Observations

In our patients, we observed a cognitive and behavior decline, exposed to significant RT doses, 30–65 Gy, similar to what was typically observed in sVAD (dysexecutive functions, apathy, and gait alterations), but with a more rapid onset and with an overwhelming effect. The observed neuropsychological deterioration was comparable with the observed increase in white matter hyper-intensities on the T2-weighted images, markedly on the periventricular regions, in the internal capsule, the paraventricular frontal and basal ganglia areas, and much more evident in the patients exposed to 45–65 Gy. The interruption of the frontal subcortical loops, which is continuously seen in SVD brain disease and sVAD, occurs in RT; the time of onset and speediness of occurrence is higher than the usual progression of sVAD, as it has been described in many different works and reviews [12,17,43]. It seems quite interesting that the same neural networks (frontal-basal ganglia-subthalamus and parietal cortex) participate in walking and gait control as well [9,12,14,20].

## 3. Radiotherapy Effects

Our results were concordant with the RT-delayed onset consequences in the brain of the patients who undergo it.

A few autopsy studies demonstrated that RT drove to diffuse myelin destruction, with the relative preservation of axons and large blood vessels [43]. The volume of brain receiving ≥12 Gy has been shown to correlate with the incidence of radiation necrosis and asymptomatic radiologic changes, and the presence of comorbid vascular risk factors (e.g., diabetes) might potentiate it [44,45,46].

Three different hypotheses were claimed out [47] to explain radiotherapy harm in brain tissue:

1. Possible pro-oxidative stress and inflammatory induction by radiotherapy;
2. Possible effect on matrix metalloproteinases and extracellular matrix in the brain;
3. Possible effect on brain angiogenesis.

Another theory of radiotherapy-induced brain damage should be mentioned, which is well known, sufficiently proven, even if not universally accepted. The human brain observational studies together with many animal experiments are entirely in agreement with the fact that there is a constant area of sufferance in the brain which undergoes RT, the so-called subventricular zone (SVZ) [48,49,50,51,52,53,54,55,56]. The SVZ area is fundamental in new-born and young animals, but has a specific role in the adult brain too; the SZV is the area where neurogenesis occurs in the adult mammalian brains, associated with some parts of the lateral walls of the ventricles, the dentate gyrus of the hippocampus and the olfactory bulb [57]. Human SVZ could promote glial precursors, towards the basal forebrain, throughout the white matter of the human brain [57], which seems to contribute to the postnatal myelination of the white matter of the frontal lobes [56,57], and RT could somehow alter the neurogenic microenvironment of the white matter of the frontal regions [55,56]. In this region, there is a proven activation of the local microglia, with a constant increase in IL-6, which seems to block the neuronal differentiation and decrease oligodendroglial proliferation [55,56,57,58,59,60,61,62]. The most vulnerable regions for cognitive radiation damages were outlined in different studies, and included the corpus callosum, left frontal white matter, right temporal lobe, bilateral hippocampi, subventricular zone, and cerebellum [63].

Oxidative stress and inflammation are the suspected crucial pathways that lead to radiation-induced brain injury [64,65,66,67,68,69]. A marked elevation of COX-1/-2 activity and prostaglandin E2 (PGE2) synthesis occurs in the brain following ionizing radiation; this evidence is in favor of a general CNS inflammation. In fact, in the CNS after RT, there is an up-regulation of different pro-inflammatory mediators, including tumor necrosis factor-α (TNF-α), IL-1β, IL-6, inducible nitric oxide synthase (iNOS), intercellular adhesion molecule-1 (ICAM-1), and matrix metalloproteinase- 9 (MMP-9) [70,71], among others. Enhanced adhesion molecules, such as ICAM-1, vascular cell adhesion molecule-1 (VCAM-1), and E-selectin, were observed in irradiated brains [64,65,68,69]. The rapid induction of the gene expressions of the pro-inflammatory cytokines, such as TNF-α and IL-1β, in response to radiation, was identified [64,68]. Moreover, a significant and marked up-regulation of mRNA and protein expression of pro-inflammatory mediators, including TNF-α, IL-1β, and monocyte chemoattractant protein-1 (MCP-1), was observed in the hippocampal and cortical regions isolated from irradiated brains. Interestingly, cytokine expression was regionally specific since TNF-α levels were significantly elevated in the cortex compared to the hippocampus, and IL-1β levels were elevated in the hippocampus compared to cortical samples. A series of electrophoretic mobility shift assays (EMSAs) also demonstrated that the whole-brain radiation significantly increased pro-oxidative and pro-inflammatory transcription factors, including AP-1, NF-κB, and CREB [72,73].

In vitro and in vivo studies showed that whole-brain radiation strongly activates the microglia in the general pro-inflammatory response [73,74]. Therefore, oxidative stress-mediated inflammation is one of the significant consequences of whole-brain radiation and plays a pivotal role in subsequent radiation-induced tissue injury to a healthy brain.

Radiotherapy disrupts the blood–brain barrier (BBB), altering the functional structure of the brain’s microvasculature and increasing its permeability [75,76,77,78,79,80,81]. The most intriguing aspect is that RT seems to damage healthy capillaries rather than those inside the tumor [78]. This was even more true in response to interstitial brachytherapy [79,80].

There is a very well accepted and proven mechanism by which BBB disruption occurs after RT. However, it seems possible that RT has specific targets, such as the extracellular matrix (ECM) proteins and proteoglycans, including collagens, laminin, fibronectin, and tenascin [82], which are the major constituents of the BBB [82,83]. The most sensitive family of the ECM subgroups to RT are the matrix metalloproteinases (MMPs), a large family of ECM-degrading enzymes [84,85,86]. MMPs are partitioned into gelatinases (MMP-2 and -9), collagenases (MMP-1, -8, -13, and -18), stromelysins (MMP-3, -10, and -11), matrilysins (MMP-7 and -26), metalloelastase (MMP-12), and membrane-type (MT) MMPs (MMP-14, -15, -16, -17, -24, and -25) [84,87]. In this case, damage to MMP-2 and MMP-9 can damage collagen type IV, which is essential for maintaining BBB integrity [85]. Moreover, the MMP activity is regulated by endogenous tissue inhibitors of metalloproteinases (TIMPs) [84,85,87]. For example, TIMP-1 inhibits MMP9 activity by forming a specific complex with MMP-9, whereas TIMP-2 bounds MMP-2 [88,89,90]. Consequently, a favorable balance of MMPs/TIMPs system maintains the BBB integrity [85,91]. This imbalance has been documented in different tissues, among them the brain, lung, thorax [92,93], prostate and rectal mucosa [94], with the overexpression of MMP-2 and MMP-9, but without the concomitant activation of TIMP, i.e., TIMP-2, [95], therefore making possible only the proteolytic activity. This activity was documented in specific brain cell types, i.e., astrocytes, endothelium, and oligodendrocytes [96,97], where RT induces an overwhelming promotion of MMP and dysregulates TIMP, exerting a generalized proteolytic effect [47].

One potential experimental approach is to administer a series of pharmacological agents that selectively inhibit MMPs by different mechanisms of action, including minocycline, simvastatin, AG3340, DPC-A37668, GM6001, PD166793, and Ro-31–9790 [98,99,100], anti-inflammatory [101], anti-oxidants [102] to animal models of whole-brain radiation therapy. However, results are not definite at all [47].

## 4. Radiotherapy Damage and Small Vessel Disease: Common Points

Between several different hypotheses of late radiation-induced brain injury, the vascular hypothesis could be a consequence of white matter necrosis.

In the RT brain, many vascular changes were observed: vessel wall thickening, vessel dilation, endothelial cell nuclear enlargement, capillary rarefaction, and general tissue hypoxia [12]. As reported above, besides the vascular hypothesis, oligodendrocytes, astrocytes, and microglia’s pro-inflammatory responses have been described [43,103]. The consequence is the diffuse sufferance of the white matter, comparable to that seen in subcortical vascular dementia [104]. Apart from the apparent necrotic region, induced by RT damage, the immunohistochemical analysis of the brains which undergo to RT found a substantial decrease in myelin-basic protein-positive fibers in radiated areas, with a moderate lessening of the neurofilament-positive fibers, which are variously dispersed in the radiated territories. Simultaneously, there is an evident increase in the gliosis process, which is testified by an increase in the quote of glial fibrillary acidic protein-positive fibers. These findings are similar to those in clinically accelerated brain aging in diffuse subcortical ischemia [12,43,47,103].

Cerebral small vessel disease (SVD) primarily distresses the small perforating arteries, supplying the deep brain structures and the leptomeningeal space [104,105]. The small-vessel disease is significantly related to the loss of the arterial elasticity, with a significant lessening of arterial compliance, low-level functioning of the autonomic nervous system, with altered baroreflex activity [106,107,108,109,110,111]. Arteriolosclerosis extents the hypo-perfusion in the profound territories [112,113], irrigated by penetrating arteries [114,115,116,117]; its consequence is potentiated by the loss of the cholinergic integrity [118] and promoted by itself a functional disconnection from the tubero-mamillary tracts [119,120,121].

Moreover, in SVD, the perivascular spaces (PVS) were damaged [122]. They act as a brain lymphatic system [123], and their modifications induce a decrease in catabolic drainage, an amassing of catabolites and toxic substances, together with a pronounced neural starvation [123,124,125] and the interference of the blood–brain barrier integrity [126,127,128,129,130].

Thus, SVD could be simplistically summarized by two main processes, progressive hypoperfusion, triggering the incomplete ischemia of the deep white matter [131,132,133,134] associated with an inflammation of the white matter. These two biological events induce histological modifications of the white matter, very similar to those observed in the post-RT brain: a diffuse rarefaction of myelin sheaths, followed by an axonal disruption and severe astrocyte gliosis [117]. We have all the data concerning RT brains, but in SVD, something more complex occurs too: there is a complex disruption of the glymphatic system, associated (or caused) by the occlusion of the deep periventricular-draining veins [135], together with a disruption of the BBB [131,132,133].

As far as the inflammatory events proceed, either in post-RT or SVD, we can find crucial microglial activation, also promoting the apoptosis processes. There is an elevation of caspase 3 RNA, and of matrix- metalloprotease 2 (MMP-2) expression [136,137], with a marked astrocytosis at the first part of the process, and then with their death (the following part has not been described in the post-RT brain, probably due to the long-lasting observation period, typical of SVD and not of post-RT) [138,139,140,141,142]. In SVD, the astrocytic death includes a severe alteration of the precarious neurovascular coupling system, leading to its destruction [143].

The effects of the inflammation in the SVD act indirectly on the endothelium [143,144]. It is widely demonstrated that, due to an altered response to endothelium-derived nitric oxide-vasodilators [145,146], prostacyclin [147] and endothelium-derived hyperpolarizing factors (EDHF) [148], there is an accelerated mitochondrial senescence of the endothelium walls [144]. The reduction of the mitochondrial strength leads to an essential lessening of NO production, with an unrestrained production of O2 [149,150]. The pathological reduction of NO is related to the downregulation of the Rho-associated protein kinase (ROCK) [151]. ROCK interacts with ezrin, radixin, and moesin, also known as the ERM proteins, that are fundamental for membrane integrity and the leukocyte adhesion molecules’ coordination, with the significant consequence for inflammation response, for endothelium integrity, and brain barrier integrity [152,153,154,155,156], also helped by the regulation of VE-cadherins [157,158,159].

The strong aftereffects of mitochondrial senescence, oxidative damage on endothelium, and brain barrier induce a cascade of potentiating events on inflammation [159]. All the principal mechanisms of inflammation, apoptosis, necroptosis, Wallerian degeneration, demyelination, astrocytosis, and microglial activation have been documented in SVD [160,161,162]. The loss of plasma membrane integrity is involved in a peculiar phenomenon, observed in SVD, called necroptosis. Necroptosis is different from apoptosis because it is programmed cell death, not involving caspase but the loss of plasma membrane integrity, through the receptor-interacting serine/threonine-protein kinase (RIPK-1) and the mixed lineage kinase domain-like (MLKL) [163,164,165]. Wallerian degeneration also occurs in SVD, which is shared with post-RT consequences [117].

Inflammation for SVD has been evoked as a priming effect, even at a distance, in the so-called microbiome–gut-brain axis [166,167,168,169,170,171]. Age is the main dominant promoting factor that leads to a series of altered responses to inflammation and oxidative process in the brain; SVD is a potentiated aging process [117,143]. Recently, neurodegeneration–inflammation–aging has been defined as “inflammaging” [172,173,174,175,176]. The loss of oxidative repair process in SVD leads to the constant activation of the glial cells [176], which endorses telomere shortening, frequently observed in many neurodegenerative conditions [177].

Oxidative stress is the critical mediator of the cascade of events derived from chronic or global hypoxia in different animal models [178]. The reduction of NO bioavailability leads to lessened anti-inflammatory properties, which promotes other oxidant molecules, accentuating vascular dysfunction, damage to proteins and DNA, and the loss of modulation of Rho-kinase activity [179], with the consequent decrease in vascular tone control [180,181,182], therefore disabling the neurovascular coupling [183,184,185].

Either in animal models, in SVD or in post-RT brain damage, ROS-mediated ischemic injuries have been described.

Nox2-NADPH oxidase is a crucial mediator of ischemic brain injury leading to BBB disruption and the dysfunction of larger pial arteries; a primary confirmation of this aspect is that Nox2-deficient mice have less brain injury after focal ischemia [186].

Potential treatment strategies for SVD and post-radiotherapy damage might include those that target antioxidant effects for the endothelium of small cerebral vessels [187] and the BBB [188,189]. Given NO bioavailability is impaired in SVD, NO donors could be useful in releasing the functioning endothelium of small vessel disease, limited by their susceptibility to tolerance development [187,190,191]. The apparent strategy, like the administration of potent antioxidants such as Vitamins C and E, has shown to be beneficial for vascular function in several experimental and small clinical trials [192,193,194]. Disappointingly, the results of large clinical trials of antioxidant supplementation have largely failed to show any benefit [195,196]. The ROS scavenger tempol is cell-permeable and has been used in experimental studies [197,198], as well as edavarone (O2-scavenger) [199]. Problems derived from NADPH oxidase activity, in particular its primary contributor, Nox2. Nevertheless, this isoform is also expressed in phagocytic immune cells; thus, it can be argued that prolonged selective therapies could help to prevent SVD, but invariably lead to an immunosuppression condition, as well as to many other side effects derived by different other Nox oxidases [200,201,202,203,204,205,206,207,208,209,210].

## 5. Conclusions

From what was reported above, and from our data, it seems quite evident that many mechanisms can be evoked to explain the mechanism through which radiotherapy acts, but what remains quite evident is that the most critical damage target is the small vessel arbor. Together with the small vessels, the other main targets of RT, outside the tumor mass are the BBB and the white matter areas. The active mechanisms that RT drives are similar to those observed in SVD [211,212]. What impresses on the active site of RT brain damage is the white matter areas, particularly the internal capsule, basal ganglia, caudate, hippocampus, and subventricular zone. In all the cases, radiation damage inside the brain mainly focuses on the cortical–subcortical frontal loops, which integrate and process the flow of information from the cortical areas, where executive functions are “elaborated” and prepared, towards the thalamus, subthalamus, and cerebellum, where they were continuously refined and executed [213,214]. Cerebral atrophy with diffuse demyelination and spongiform changes in the white matter is seen pathologically [215]. This typical disposition clarifies the cognitive and behavioral subcortical frontal and white matter alterations observed in different patients.

These data are entirely per the results presented from our study reported herein. The main difference between pure SVD and post-RT damage is the speediness of damage accumulation, typical of RT damage, and it is strongly related to the total amount of given radiation. Putative mechanisms that can be taken into account are oxidative stress and neuro-inflammation, strongly associated with the alteration of NMDA receptor subunit composition [211]. A strong preponderance of evidence supports the hypothesis that late radiation injury in the brain is driven by acute and chronic oxidative stress and inflammatory response, generating ROS, which leads to a cascade of events, the primary of which is the damage of DNA, cell membrane bilayers, and the activation of early response transcription factors and signal transduction pathways [212].

Even in a small number of clinical trials, particularly at the time of enrollment, more precise biochemical definition strategies must refer to these therapeutic choices as future options, which will need more robust and affordable, more extensive studies.

## Figures and Tables

**Table 1:** Baseline characteristics of the Group A patients.

Group A	
Number of cases	114
Mean age	45.2 ± 3.7
Handedness Briggs and Nebes Test [42]	22.4
Educational level	17.7 + 1.2 years of school
Diagnosis	
	44 glioblastoma multiforme
	26 cerebral lymphomas (mild- or low-grade differentiation
	19 grade-2 gliomas
	8 craniopharyngiomas
	12 II-III grade-astrocytomas
	5 anaplastic patterns.

**Table 2:** Radiotherapy amounts.

	Dose of Radiation	Histology	CT
26 patients	<30 Gy	8 craniopharingiomas 10 grade 2 gliomas 8 grade 2 astrocytomas	Any
44 patients	30–45 Gy	18 glioblastoma multiforme 10 cerebral lymphoma 9 grade 2 gliomas 4 grade 3 astrocytomas 3 anaplastic pattern	6 MTX 6 Rituximab 6 Temozolamide
44 patients	45–65 Gy	26 glioblastoma multiforme 16 cerebral lymphomas 2 anaplastic patterns	10 MTX 16 Rituximab 26 Temozolamide

**Table 3:** A synopsis of the scores obtained by the two groups at baseline (Group A, before radiotherapy (RT)).

Variables	Group A	Group B	F CHI2 Value	DF	*p*-Value
MMSE	28.3 ± 2.2	27.3 ± 2.3	0.97	2.88	0.56
Phon. Fluency (items produced)	45.6 ± 2.4	39.3 ± 3.5	0.98	2.34	0.67
Sem. Fluency (items produced)	32.1 ± 2.5	27.3 ± 2.3	0.87	2.45	<0.05
Mental calculation (correct/15)	11.4 ± 2.3	9.8 ± 1.3	0.89	2.34	0.78
Analogies (correct/26)	24.5 ± 5.6	19.3 ± 2.4	0.89	2.45	<0.01
Digit span forward	6.7 ± 2.3	4.5 ± 2.1	0.78	2.36	<0.01
Digit span backward	5.9 ± 1.2	4.1 ± 1.2	0.87	2.56	<0.01
BEHAVE-AD	4.5 ± 7.6	3.7 ± 0.3	0.89	2.34	0.98
AES-S	10.3 ± 1.1	12.1 ± 5.3	0.86	2.55	0.87
AES-C	9.2 ± 2.1	13.9 ± 1.2	0.98	2.33	0.89

**Table 4:** A synopsis of the scores obtained by the patients of Group A who underwent to a total dose of <30 cGy, at 12 months.

Variables	Group A Pts < 30 Gy	Group B	F CHI2 Value	DF	*p*-Value
MMSE	25.9 ± 1.7	26.34 ± 2.2	0.97	2.88	0.56
Phon. Fluency (items produced)	40.1 ± 2.2	31.67 ± 2.4	0.98	2.45	<0.01
Sem. Fluency (items produced)	34.5 ± 1.23	24.4 ± 1.4	0.81	2.45	<0.01
Mental calculation (correct/15)	12.3 ± 1.4	11.8 ± 1.3	0.84	2.34	0.78
Analogies (correct/26)	19.2 ± 1.1	24.5 ± 5.6	0.85	2.53	<0.05
Digit span forward	6.7 ± 0.4	6.8 ± 2.3	0.78	2.36	0.67
Digit span backward	4.65 ± 0.7	6.1 ± 0.3	0.85	2.52	<0.05
BEHAVE-AD	3.1 ± 1.4	2.7 ± 0.4	0.89	2.37	0.98
AES-S	9.5 ± 1.4	12.1 ± 0.4	0.86	2.55	<0.01
AES-C	12.1 ± 1.4	15.9 ± 4.3	0.98	2.33	<0.05
Tinetti Gait	13.1 ± 0.1	10.1 ± 0.1	0.89	2.37	0.98
Tinetti Equilibrium	10.2 ± 1.1	10.2 ± 1.1	0.82	2.12	0.45
Tinetti Total score	23.3 ± 1.2	20.3 ± 1.2	0.84	2.01	0.76

**Table 5:** A synopsis of the scores obtained by the patients of Group A who underwent to a total dose of 30–45 Gy, at 12 months.

Variables	Group A Pts 30–45 Gy	Group B	F CHI2 Value	DF	*p*-Value
MMSE	26.1 ± 1.2	26.34 ± 2.2	0.78	2.88	0.59
Phon. Fluency (items produced)	28.3 ± 2.6	31.67 ± 2.4	0.98	2.45	<0.05
Sem. Fluency (items produced)	26.7 ± 2.9	24.4 ± 1.4	0.81	2.45	<0.05
Mental calculation (correct/15)	6.7 ± 1.2	11.8 ± 1.3	0.84	2.34	0.78
Analogies (correct/26)	17.9 ± 4.1	24.5 ± 5.6	0.85	2.53	<0.01
Digit span forward	4.9 ± 1.4	6.8 ± 2.3	0.77	2.36	<0.05
Digit span backward	4.1 ± 2.2	6.1 ± 0.3	0.85	2.52	<0.05
BEHAVE-AD	5.84 ± 3.89	2.7 ± 0.4	0.89	2.78	<0.05
AES-S	19.4 ± 1.4	12.1 ± 0.4	0.86	2.54	<0.01
AES-C	21.5 ± 1.4	15.9 ± 4.34	0.98	2.31	<0.01
Tinetti Gait	12.4 ± 0.2	10.1 ± 0.1	0.89	2.37	0.98
Tinetti Equilibrium	10.8 ± 0.7	10.2 ± 1.1	0.82	2.12	0.45
Tinetti Total score	23.2 ± 0.3	20.3 ± 1.2	0.84	2.01	0.76

**Table 6:** A synopsis of the scores obtained by the patients of Group A who underwent to a total dose 45–65 Gy, at 12 months.

Variables	Group A Pts 45–65 Gy	Group B	F CHI2 Value	DF	*p*-Value
MMSE	19.0 ± 2.7	26.34 ± 2.2	0.78	2.88	<0.01
Phon. Fluency (items produced)	14.5 ± 1.7	31.67 ± 2.4	0.98	2.45	<0.01
Sem. Fluency (items produced)	13.2 ± 1.5	24.4 ± 1.4	0.81	2.45	<0.01
Mental calculation (correct/15)	4.1 ± 0.2	11.8 ± 1.3	0.84	2.31	<0.01
Analogies (correct/26)	12.2 ± 0.6	24.5 ± 5.6	0.88	2.35	<0.01
Digit span forward	4.0 ± 0.8	6.8 ± 2.3	0.77	2.36	<0.05
Digit span backward	3.6 ± 0.3	6.1 ± 0.3	0.85	2.52	<0.01
BEHAVE-AD	5.7 ± 4.7	2.7 ± 0.4	0.89	2.78	<0.05
AES-S	21.8 ± 3.4	12.1 ± 0.4	0.86	2.54	<0.01
AES-C	25.2 ± 2.2	15.9 ± 4.34	0.98	2.31	<0.01
Tinetti Gait	9.1 ± 0.1	10.1 ± 0.1	0.89	2.37	0.067
Tinetti Equilibrium	7.2 ± 1.4	10.2 ± 1.1	0.82	2.12	<0.05
Tinetti Total Score	15.3 ± 1.5	20.3 ± 1.2	0.84	2.01	<0.05

**Table 7:** Marginal homogeneity test for multiple comparison (for the first row: the first *p*-value is the comparison of 45–65 Gy vs. <30 Gy; the second is the comparison of 30–45 Gy vs. <30 Gy; for the second row, the *p*-value is the comparison of 30–45 Gy vs. 45–65 Gy).

Variables	RT < 30 Gy	RT 30–45 Gy	RT 45–65 Gy
MMSE	24.2 ± 1.6	21.3 ± 1.2	12.1 ± 2.7
	*p* < 0.05; *p* < 0.01	*p* < 0.01	
Phon. Fluency	35.2 ± 2.2	19.7 ± 2.6	7.5 ± 1.7
	*p* < 0.01; *p* < 0.01	*p* < 0.01	
Sem. Fluency (items produced)	29.1 ± 1.3	15.3 ± 2.9	9.2 ± 1.5
	*p* < 0.01; *p* < 0.01	*p* < 0.01	
Mental calculation (correct/15)	10.5 ± 1.4	5.2 ± 1.2	2.3 ± 0.2
	*p* < 0.01; *p* < 0.01	*p* < 0.01	
Analogies (correct/26)	18.6 ± 1.1	9.7 ± 4.1	6.2 ± 0.6
	*p* < 0.01; *p* < 0.01	*p* < 0.05	
Digit span forward	5.1 ± 0.4	3.3± 1.4	2.7 ± 0.8
	*p* < 0.05; *p* < 0.01	*p* < 0.05	
Digit span backward	4.3 ± 0.7	2.0 ± 2.2	1.6 ± 0.3
	*p* < 0.01; *p* < 0.01	*p* < 0.01	
BEHAVE-AD	4.8 ± 1.4	8.7 ± 3.8	10.6 ± 4.5
	*p* < 0.01; *p* < 0.01	*p* < 0.01	
AES-S	12.6 ± 1.4	27.5 ± 1.4	39.8 ± 3.4
	*p* < 0.01; *p* < 0.01	*p* < 0.01	
AES-C	17.2 ± 1.4	30.8 ± 1.4	45.2 ± 2.2
	*p* < 0.01; *p* < 0.01	*p* < 0.01	
Tinetti Equilibrium	12.0 ± 0.1	9.13 ± 0.4	6.1 ± 0.8
	*p* < 0.05; *p* < 0.01	*p* < 0.01	
Tinetti Gait	9.2 ± 1.1	7.5 ± 1.2	2.2 ± 1.1
	*p* < 0.05; *p* < 0.01	*p* < 0.01	
Tinetti Total Score	21.2 ± 1.2	16.38 ± 1.3	8.4 ± 1.2
	*p* < 0.05; *p* < 0.01	*p* < 0.01	
